# Prognostic model for survival of patients with abdominal aortic aneurysms treated with endovascular aneurysm repair

**DOI:** 10.1038/s41598-022-24060-5

**Published:** 2022-11-15

**Authors:** Lorenz Meuli, Alexander Zimmermann, Anna-Leonie Menges, Sandra Stefanikova, Benedikt Reutersberg, Vladimir Makaloski

**Affiliations:** 1 Department for Vascular Surgery, University Hospital Zurich, Zurich, Switzerland; 2 Department for Vascular Surgery, Inselspital, Bern University Hospital, Bern, Switzerland

**Keywords:** Risk factors, Aneurysm, Aortic diseases

## Abstract

The role of endovascular aneurysm repair (EVAR) in patients with asymptomatic abdominal aortic aneurysm (AAA) who are unfit for open surgical repair has been questioned. The impending risk of aneurysm rupture, the risk of elective repair, and the life expectancy must be balanced when considering elective AAA repair. This retrospective observational cohort study included all consecutive patients treated with standard EVAR for AAA at a referral centre between 2001 and 2020. A previously published predictive model for survival after EVAR in patients treated between 2001 and 2012 was temporally validated using patients treated at the same institution between 2013 and 2020 and updated using the overall cohort. 558 patients (91.2% males, mean age 74.9 years) were included. Older age, lower eGFR, and COPD were independent predictors for impaired survival. A risk score showed good discrimination between four risk groups (Harrel’s C = 0.70). The 5-years survival probabilities were only 40% in “high-risk” patients, 68% in “moderate-to-high-risk” patients, 83% in “low-to-moderate-risk”, and 89% in “low-risk” patients. Low-risk patients with a favourable life expectancy are likely to benefit from EVAR, while high-risk patients with a short life expectancy may not benefit from EVAR at the current diameter threshold.

## Introduction

Abdominal aortic aneurysm (AAA) rupture is one of the leading causes of death in men over 65 years of age^1,2^. To decrease the mortality from AAA, rupture can be prevented by elective aneurysm repair^3–5^. The risk of AAA rupture increases with increasing diameter. Current treatment guidelines recommend that small and asymptomatic AAAs are observed as the risk of rupture is very low, while large aneurysms (≥ 5.5 cm in men; ≥ 5 cm in women) should be treated surgically^6–8^. Today, endovascular aneurysm repair (EVAR) is the preferred and less invasive alternative to open repair, especially in elderly patients with concomitant diseases^9–11^. However, data is conflicting on whether frail patients should be treated with EVAR or not treated surgically at all^11–13^. The EVAR-2 trial, in which patients deemed unfit for open repair were randomised to EVAR or no surgical treatment, showed no overall survival benefit for EVAR^12^. Nevertheless, about 20% of patients randomised to no surgical treatment were still alive after 8 years. These patients may have been the fittest among those enrolled in the study and remained at risk of AAA rupture for a long time^11^. Noronen et al. showed in a Finnish cohort that AAA rupture was the most common cause of death in patients with an AAA ≥ 5.5 cm in diameter who did not meet surgical requirements^13^. Today, elective EVAR can be performed with a mortality of < 1%^14^. Accurate identification and assessment of the risk profile of patients within the cohort of presumed physically unfit patients is critical. Overall mortality could be reduced by performing EVAR in the fittest patients with large AAAs, and costs could be reduced by not performing EVAR in patients with the highest risk profiles but smaller aneurysms.

The revised 2020 UK National Institute for Health and Care Excellence (NICE) guidelines on the diagnosis and management of AAA states that EVAR *or* conservative management should be considered in patients with unruptured AAA that meet the treatment indication criteria but have medical comorbidities that contraindicate open surgical repair^7^. Thereby, the NICE guidelines clearly question the role of EVAR as a treatment option in patients with asymptomatic AAA. In any case, the impending risk of aneurysm rupture, the risk of elective repair, and the life expectancy are crucial for the discussion of treatment options in patients with asymptomatic AAA.

### Risk of rupture

The natural history of large AAA in patients without treatment has been studied in several cohorts^13,15–17^. Parkinson et al.^15^ summarised the data published up to January 2014 and included 11 studies on a total of 1514 patients with 347 ruptures. A more recently published study of 3248 patients with intact AAA prospectively registered in Northern California over a 17-years period found lower annual rupture rates than Parkinson et al. for AAA with diameters 5.5–6.0 cm with 2.2% compared to the previously reported 3.5%^15,16^. But higher annual rupture rates for AAA with diameters 6.1–7.0 cm with 6.0% compared to 4.1% and for AAA with diameters > 7 cm with 18.4% compared to 6.3%^15,16^.

### Risk of repair

Elective AAA repair carries a perioperative risk of mortality of 0.9% to approximately 5%^14,18^. Several risk predictive models for perioperative mortality have been presented^19–23^. The most recently published scoring scheme by Eslami et al. used the the Vascular Study Group of New England (VSGNE) database and included aneurysm diameter, neck length, and level of clamp placement as well as age, type of repair (open repair vs. EVAR), female sex, myocardial disease, congestive heart failure, chronic obstructive pulmonary disease (COPD), and, estimated glomerular filtration rate (eGFR) to predict peri-operative mortality. It was superior to the other models in terms of discrimination ability and was robust on external validation^19–21^. The use of this model by surgeons has been recommended to assist in making informed decisions and recommendations about aneurysm repair by the national academic society for vascular surgery (SVS)^8^.

### Life expectancy

Survival information of patients with AAA is available from several RCTs and from large registry data on patients that were surgically treated for their AAA^24–27^. The median survival after EVAR ranges from only 3 to 8 years in the UK EVAR trials^12,25^. Several studies have identified factors that are associated with impaired survival in patients classified as unfit for open aneurysm repair^21,28–31^. At a tertiary referral centre for vascular surgery in Bern, Switzerland, lower eGFR, COPD, and age were identified as independent risk factors for mortality after EVAR in patients treated between January 2001 and December 2012 for asymptomatic AAA^30^.

The aim of this study was to perform a temporal validation of this predictive model using more recent data from this institution.

## Materials and methods

This retrospective observational cohort study includes all consecutive patients treated with elective EVAR for asymptomatic infrarenal AAA between January 2001 and December 2020 at a tertiary aortic referral centre in Switzerland. Patients with complex aneurysm repair including fenestrated, branched, or parallel grafts were excluded from this study. Further, all patients treated for symptomatic or ruptured aneurysms as well as for other indications like penetrating aortic ulcers were excluded.

The study comprises of two cohorts: Patients treated between January 2001 and December 2012 ("Cohort A"), results of these patients have previously been published^30^. Patients treated between January 2013 and December 2020 constitute "Cohort B", results of this cohort have not yet been presented. Survival information was obtained from the hospital database and verified by a cross-sectional telephone survey of patients’ general practitioner and families in January 2017 for Cohort A and in April 2021 for Cohort B. To avoid attrition bias, survival information was right censored at these two dates respectively. Completeness of follow-up information was reported using the Follow-up Index (FUI)^32^.

This study was conducted according to the Principles of the Declaration of Helsinki and reported in adherence to the TRIPOD- Statement (Transparent Reporting of a multivariable prediction model for Individual Prognosis Or Diagnosis)^33^.

The study was approved by the regional ethics committee of swissethics (Kantonale Ethikkommission Bern, Switzerland, BASEC-ID: 2022-00489).

Waiver of informed consent was granted in accordance with Article 34 of the Swiss Human Research Act by the regional ethics committee of swissethics (Kantonale Ethikkommission Bern, Switzerland).

### Statistical analysis

The primary outcome of this study was overall survival. Baseline characteristics for both cohorts were summarized and compared. Dyslipidaemia, diabetes mellitus and arterial hypertension were coded if patients had a diagnosis or if patients had statins for dyslipidaemia, antidiabetics or antihypertensives (including beta-blockers) in their medication at discharge from hospital.

Continuous variables were summarized by mean and standard deviation if normally distributed or by median and interquartile range if skewed. Normality was visually inspected using histograms and tested using the Shapiro–Wilk test. Factor variables were compared by Chi2-test, continuous variables by student's t-test if normally distributed or by the Kruskal–Wallis rank test if skewed. All statistical analyses were performed using R Studio (Version 3.6.3) on MacOS version 11.4.

#### Predictor selection

The variable selection process for the original model on Cohort A has been described in detail^30^. In short, a pre-selection of variables was conducted based on a literature review to avoid a complete data-driven variable selection. Thereafter, least absolute shrinkage and selection operator (LASSO) with tenfold cross-validation technique was used for variable selection of the predictive model. The identified factors were eGFR, age, and COPD.

#### Cox proportional hazard model

The original Cox predictive model included the variables age, eGFR, and COPD as a binary variable. Discrimination ability of this model was tested on Cohort B as a temporal validation using Harrell’s concordance statistics *C*. Thereafter, the model was updated on the overall cohort (Cohort A + B). Discrimination ability of the final updated model was tested using 10,000 bootstrap samples of the overall cohort. The concordance statistics and the coefficients with corresponding 95% confidence intervals (95% CI) were obtained from the bootstrap samples using the percentile method. The proportional hazards assumption was tested using scaled Schoenfeld residuals for each of the Cox models.

#### Predictive score

The beta-coefficients of the final model were used for constructing the risk score. Age and eGFR were categorized into four groups. The groups were formed using quartiles for orientation but then rounded to the next clinically convenient integer. Coefficients for age groups, eGFR groups and COPD were multiplied by 10 and rounded to the nearest integers to compile the score. The risk score was cut into four risk groups at the quartiles of the point scale.

Kaplan–Meier survival curves were plotted for both cohorts and compared using the Logrank test. Further, the overall cohort was stratified by each of the predicting variables and the risk score groups. Survival curves were plotted for the stratified cohort.

## Results

**Figure 1:**
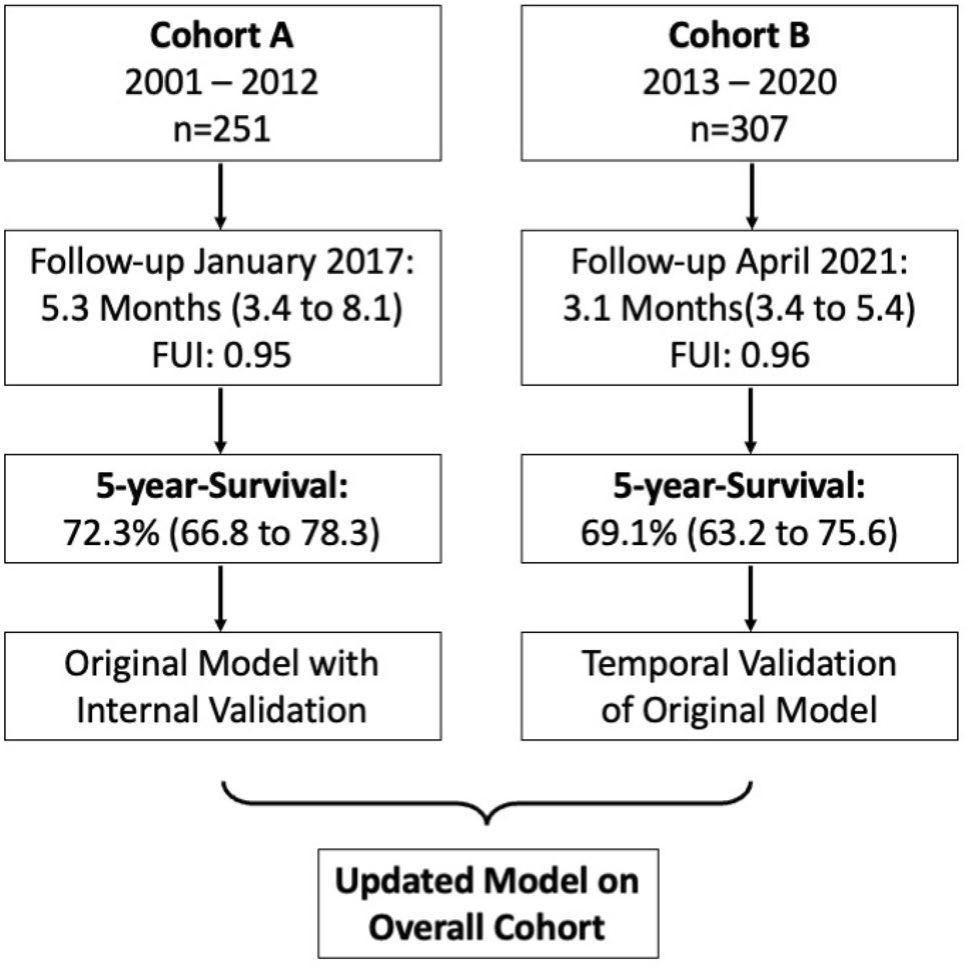
Study Cohort. Follow-up period is reported by median and quartiles (Q1–Q3). 5 years-Survival is reported with median and 95% confidence intervals. FUI = Follow-up Index.

From January 2001 to December 2020 a total of 558 patients underwent elective EVAR for non-ruptured AAA at a tertiary aortic referral centre in Switzerland. In Cohort A, period 2001–2012, 251 patients were treated. In Cohort B, period 2013–2020, 307 patients were treated, see Fig. 1.

Table 1 shows the baseline characteristics of the patients prior to EVAR for both cohorts. Patients treated after 2012 were healthier in terms of the measured comorbidities: They had better kidney function (median eGFR 70.5 versus 60.9 ml/min/1.73m^2^, *P* < 0.001), were less likely to have COPD (20.3% vs. 28.4%, *P* = 0.025), and fewer had PAD (no PAD in 85.6% versus 70.9%, *P* < 0.001). The proportion of patients with dyslipidaemia was higher in Cohort B (90.2 vs. 77.7%, *P* < 0.001). Multivariable Cox proportional hazard models were calculated for both cohorts for all available variables previously identified as associated with survival to allow inclusion of these data in future studies (Supplementary Table 1).

**Table 1:** Baseline Characteristics.

Variable	Cohort A 2001–2012	Cohort B 2013–2020	*P*-value
	n = 251	n = 307	
Age, median years (IQR)	76.0 (69.0–80)	75.7 (69.8–80.7)	.393
Male sex	234 (93.2)	275 (89.6)	.130
Arterial hypertension	210 (83.7)	252 (85.1)	.636
Missing	-	11	
Diabetes mellitus	50 (19.9)	59 (19.3)	.850
Missing	1	1	
Dyslipidemia	195 (77.7)	276 (90.2)	< .001
Missing	1	1	
BMI, median (IQR)	26.9 (24.6–30.0)	27.0 (24.0–30.0)	.510
Missing	3	30	
Smoking	191 (76.4)	203 (71.0)	.156
Missing	1	21	
COPD	71 (28.4)	62 (20.3)	.025
Missing	1	2	
eGFR	60.9 (47.5–75.6)	70.5 (53.2–83.0)	< .001
Missing	1	3	
Creatinine, µmol/l	93.0 (77.8–112.0)	90.0 (77.0–110.0)	.322
Missing	1	4	
PAD, none	178 (70.9)	262 (85.6)	< .001
Fontaine stage 1	28 (11.2)	21 (6.9)	
Fontaine stage 2	24 (9.6)	23 (7.5)	
Fontaine stage 3	21 (8.4)	0	
Missing	1	1	
Coronary artery disease	135 (53.8)	175 (57.8)	.349
Missing	4	4	
Myocardial infarction	58 (23.2)	56(18.5)	.178
Missing	1	5	
Aneurysm diameter, mm	57.8 (10.7)	60.2 (11.0)	.011
Missing	9	16	

Data was complete if not stated explicitly. Continuous variables are presented by the mean and (standard deviation) if normally distributed of by median and (interquartile) range if skewed. Counts are presented with number and (percentage).

*BMI* body mass index, *COPD* chronic obstructive pulmonary disease, *eGFR* estimated glomerular filtration rate according to the “Modification of Diet in Renal Disease Study” (MDRD) equation presented as mean ml/min/1.73 m^2^ and (standard deviation), *PAD* peripheral arterial disease as clinical stage according to the Fontaine classification.

Factor variables were compared by chi2 test, continuous variables by Student's t-test if normally distributed or by the Kruskal–Wallis rank test if skewed, respectively.

The median follow-up time was 5.3 (IQR: 3.4–8.1) years in Cohort A and 3.1 (IQR: 3.4–5.4) years in Cohort B. Follow-up information was almost complete for both cohorts (FUI 0.95 and 0.96 for Cohort A and B, respectively). The overall survival at 1 and 5 years were similar in both cohorts, *P* = 0.102. One year survival was 95.2% (95% CI 92.6–97.9%) in Cohort A and 90.9% (95% CI 87.6–94.2%) in Cohort B. At 5 years, mortality was 35.9% (90 of 251 patients) in Cohort A, and 36.2% (111 of 307 patients) in Cohort B, see Tables 2, 3 and Supplementary Fig. 1.

**Table 2:** Updated model predicting overall survival.

Variable	Estimates (Overall cohort)			Bootstrap validation	
	Hazard ratio	95% CI of HR	*P*-value	Hazard ratio	95% CI of HR
Age	1.089	1.065–1.114	< .001	1.090	1.065–1.118
COPD	2.138	1.579–2.895	< .001	2.163	1.562–3.032
Kidney function	1.012	1.004–1.020	.002	1.012	1.003–1.022

**Table 3:** Model performance.

	Estimates (overall cohort)			Bootstrap validation	
	Harrell's *C*	95% CI of *C*	*P*-value	Harrell's C	95% CI of *C*
Overall model	0.701	0.656–0.745	< .001	0.701	0.656–0.744

Cox proportional hazard model on overall survival. Complete-case analysis, n = 552 of 558, missing data as presented in Table 1; number of events was 197; global test for statistical significance of the model, LR-test: *P* < .001.

COPD, chronic obstructive pulmonary disease; not having the condition served as reference group; kidney function as estimated glomerular filtration rate according to the “Modification of Diet in Renal Disease Study” equation; inversed Hazard Ratios (HR) are presented to facilitate reading and comparability to previously published data; the HR increases as the eGFR decreases and vice-versa.

### Temporal validation of the Cox model for survival after EVAR

The original model based on Cohort A was tested on Cohort B and showed moderate to good discrimination ability (*C*: 0.683, 95% CI 0.617–0.748). The discrimination ability was slightly lower than on the original Cohort A (*C*: 0.722, 95% CI 0.667–0.778).

### Updated Cox model for survival after EVAR

The model was then updated using the overall cohort. The Cox model showed good discrimination ability (*C* of 0.701, 95% CI 0.656–0.745) and was very robust on internal validation using 10,000 bootstrap samples (*C* of 0.701, 95% CI 0.656–0.744), see Table 3.

Increased age (HR 1.09, 95% CI 1.07–1.11, per year increase), the presence of COPD (HR 2.14, 95% CI 1.58–2.90), and impaired kidney function (HR 1.13, 95% CI 1.04–1.22, per 10 ml/min/1.73^2^ decrease in eGFR) were independently associated with a poor overall survival. The model specifications are presented in Table 2. The impact of these variables is visualized by the Supplementary Figs. 2–4.

### Risk score for 5-years survival after EVAR

Table 4 shows the predictive model derived from the overall cohort and based on the three variables age in groups, COPD (yes/no), and the kidney function (eGFR) according to the KDIGO classification (kidney disease: improving global outcomes). KDIGO stage G4 and G5 were merged to get a reasonable number of patients per group.

**Table 4:** Risk score.

Variable	Predictive model for 5-years survival			
	Points	Hazard ratio	95% CI of HR	
**Age in years**				
< 70 years	0	Ref.	Ref.	
70–74.9 years	9	2.34	1.43–3.82	
75–79.9 years	10	2.66	1.64–4.33	
≥ 80 years	17	5.71	3.58–9.12	
**COPD**				
Yes	7	2.10	1.54–2.86	
**KDIGO stage (eGFR in ml/min/1.73 m**^**2**^**)**				
G1 (> 90)	0	Ref.	Ref.	
G2 (60–89.9)	1	1.06	0.58–1.91	
G3a (45–59.9)	3	0.89	0.47–1.69	
G3b (30–44.9)	6	1.77	0.92–3.39	
G4/5 (< 30)	15	4.29	2.11–8.71	

Risk category	Score	1 year survival	5 years survival	
Low	≤ 8	99% (97–100%)	89% (84–95%)	
Low to moderate	9–13	99% (97–100%)	83% (76–90%)	
Moderate to high	15–18	92% (87–97%)	68% (60–79%)	
High	≥ 19	82% (75–89%)	40% (32–50%)	

Variable	Original estimates		Bootstrap validation	
	Harrell's *C* (95% CI)	*P*-value	Harrell's *C* (95% CI)	*P*-value
Risk score	0.703 (0.659–0.747)	< .001	0.703 (0.658–0.745)	< .001

Predictive score based on Cox proportional hazard model. Complete-case analysis, n = 552 of 558, missing data as presented in Table 1; number of events was 197; global test for statistical significance of the model, LR-test: *P* < .001. 1 year and 5 years survival are presented with 95% confidence interval in brackets.

*COPD* chronic obstructive pulmonary disease; not having the condition served as reference group. *eGFR* estimated glomerular filtration rate according to the “Modification of Diet in Renal Disease Study” equation and grouped according to the KDIGO classification.

The combined score of the three variables can be used to predict the expected 5 years survival probability for patients with this risk profile. The summary score was split into four risk groups. Patients with a summary score of ≥ 19 points are in the "high risk" groups and are expected to have a 5 years survival probability of only 40% (95% CI 32–50%), whereas patients with ≤ 8 points are in the "low risk" group and have an excellent 5 years survival probability of 89% (95% CI 84–95%). In between there is a "low-to-moderate-risk" (9–13 points) and "moderate-to-high-risk" (15–18 points) group with 5 years survival probabilities of 83% (95% CI 76–90%) and 68% (95% CI 60–79%), respectively. No combination results in a score of 14 points.

**Figure 2:**
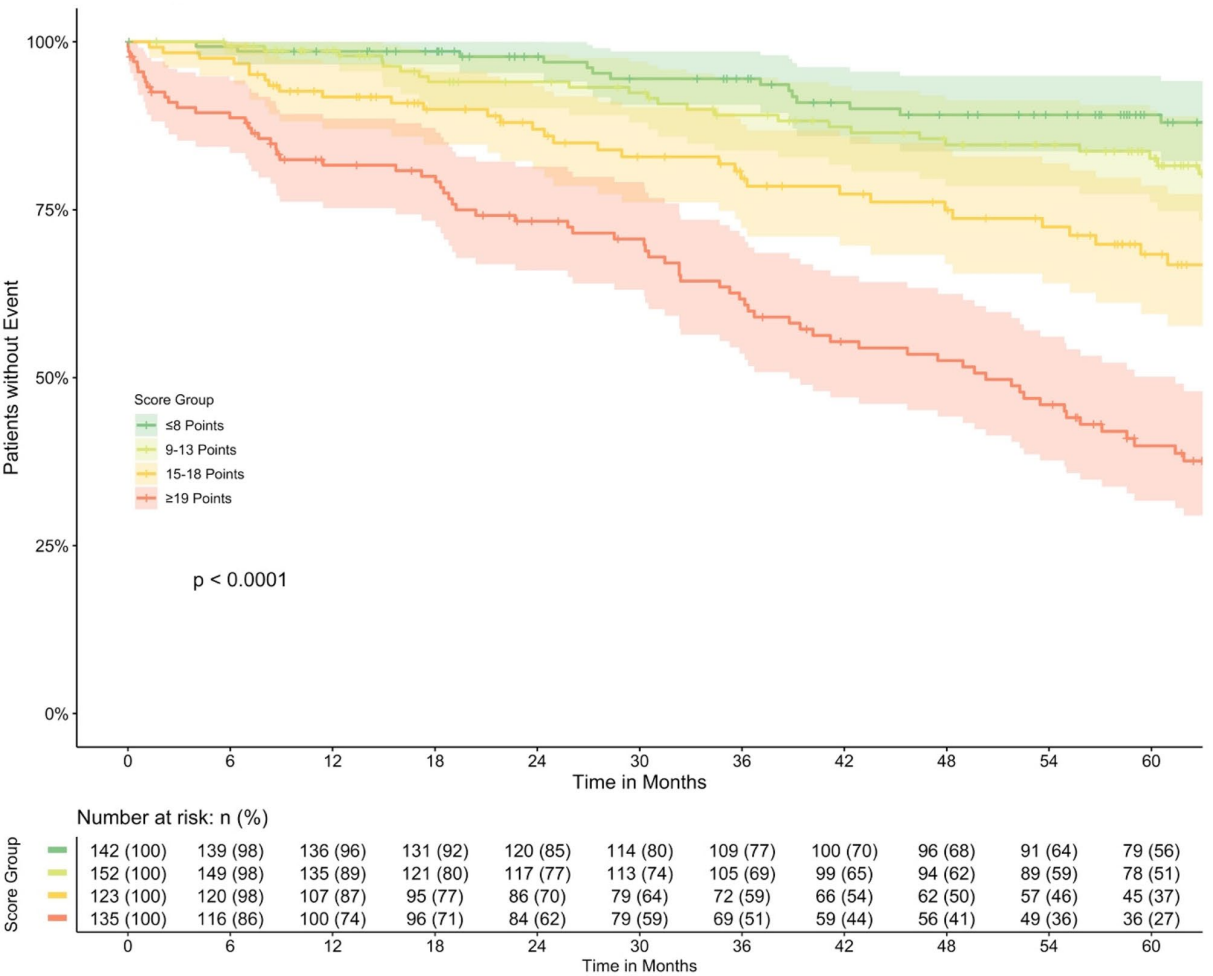
Survival Curves of Predictive Score. Kaplan–Meier Estimators with corresponding 95% confidence interval for overall survival stratified by risk score. Log-rank test was used to compare difference in survival.

**Figure 3:**
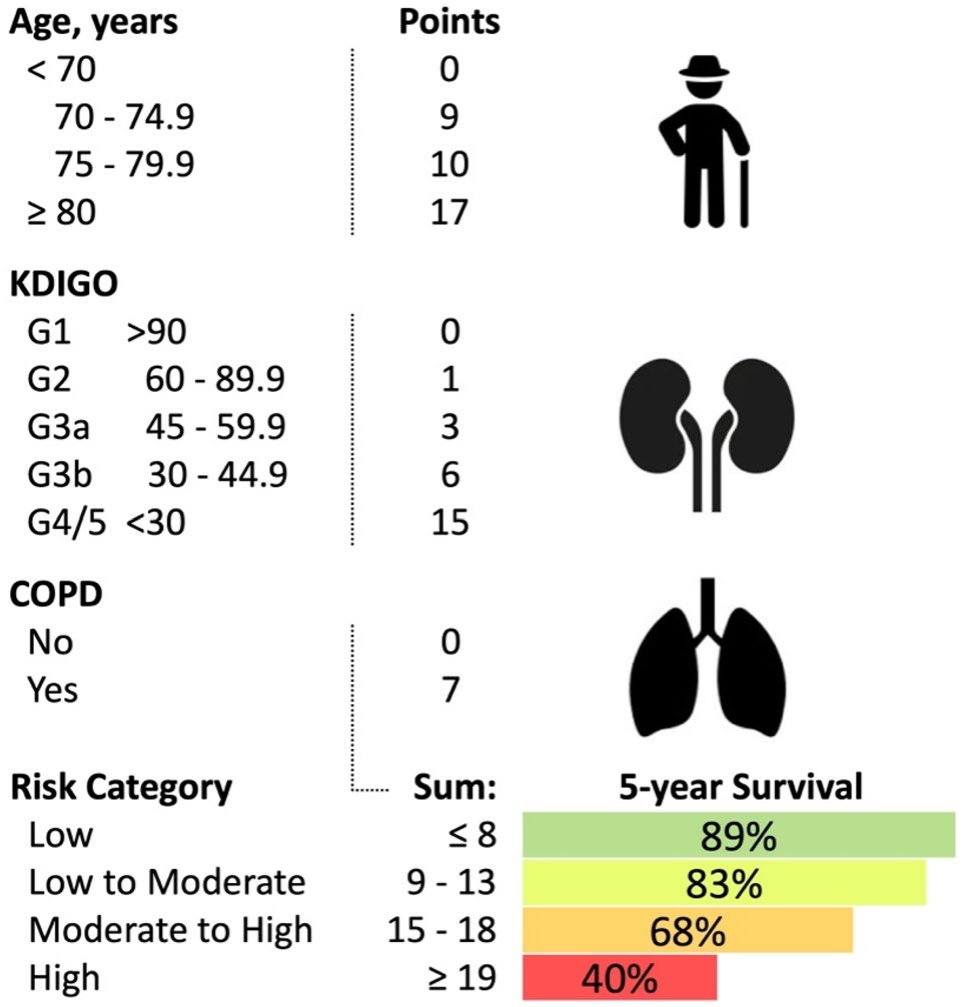
Predictive Score. Predictive score based on the Cox proportional hazard model presented in Table 4. Adding the points from Age, COPD and KDIGO gives a total score that determines the risk category. COPD: chronic obstructive pulmonary disease; KDIGO: kidney function as estimated glomerular filtration rate according to the “Modification of Diet in Renal Disease Study” equation.

The score was tested on the overall cohort and showed good discrimination ability (*C* of 0.703, 95% CI 0.659–0.747) and was very robust on the internal validation using 10,000 bootstrap samples (*C*: 0.703, 95% CI 0.658–0.745), see Table 4. Figure 2 shows the actual Kaplan–Meier estimators for survival after EVAR of the overall cohort stratified by risk score groups. Figure 3 can be used to estimate the live expectancy of patients with asymptomatic AAA based on the three predictors.

#### Examples

A 75-years-old (10 points) patient with COPD (7 points) and KDIGO 3a (3 points) is in the "high-risk" group (≥ 19 points) and has an expected 5 years survival of only 40% (32–50%).

A 81-years-old (17 points) patient without COPD (0 points) and a normal kidney function KDIGO G1 (0 points) is in the "moderate-to-high-risk" group (15–18 points) and has an expected 5 years survival of 68% (60–79%).

A 68-years-old (0 points) patient with COPD (7 points) and a moderately impaired kidney function KDIGO G3a (3 points) is in the "low-to-moderate-risk" group (9–13 points) and has an expected 5 years survival of 83% (76–90%).

## Discussion

This temporal validation study analysed patient survival after elective EVAR at a large aortic centre in Switzerland. A cohort of patients treated between 2013 and 2020 was used to test and update an existing predictive model for survival after EVAR that was built on patients treated at the same institution from 2001 to 2012. The predictive model was robust in the new cohort but performed slightly worse than in the original cohort. After updating the model, it showed good discriminatory ability and was robust in bootstrap validation for the entire cohort. Older age, COPD and impaired renal function were confirmed as independent predictors of worse survival 5 years after EVAR.

Previously published predictive model for survival after EVAR did either not include survival information > 2 years^34^, were not externally validated^35–37^ or not robust on external validation^38^, or did not show sufficiently good discrimination^39,40^.

### Life expectancy

Historical data from the EVAR trial 2 showed no benefit in overall survival in patients treated with EVAR compared to conservative management in physically frail patients with asymptomatic AAA. Nevertheless, EVAR led to a significant reduction in aneurysm related mortality. The high overall mortality in this cohort dominated the additional benefit of EVAR. Of note, the mean AAA diameter in both groups (conservative and EVAR) was 6.7 cm (SD: 1 cm)^12^. This lack of evidence from RCTs supporting the use of EVAR in frail patients has fuelled the debate about its role in this patient group. Thereby, a clear definition of frailty or the identification of predictors for life expectancy of patients with AAA are missing.

In this study, COPD, impaired kidney function and age were identified as independent parameters for a shorter life expectancy. With its limitations, this study helps to identify a subgroup of patients with AAA who may not benefit from EVAR at the current diameter threshold, but rather receive continued aneurysm diameter monitoring and best medical treatment. On the other hand, it helps to identify a subgroup of patients with AAA and a favourable risk profile for whom elective AAA treatment might become strongly recommended rather than considered. Further validation on a truly external cohort and on a larger scale is needed before the next step in this process can be taken.

### Diameter threshold

The expected remaining lifetime should be weighed against the estimated aneurysm rupture rate and the periinterventional risk. These thoughts are discussed in the current European Society for Vascular Surgery (ESVS) clinical practice guidelines where a sliding scale for assessing fitness for repair as the aneurysm enlarges is mentioned^6^. However, apart from a recommendation for referral to specialists in case of comorbidities, no clear recommendations are formulated.

In the daily routine, the decision if a patient’s AAA should be treated is mainly based on its diameter and the general health condition. Usually, a threshold of 5.5 cm diameter in men and 5.0 cm in women is used for decision-making whereas frailty is often a rather vague and non-specific overall impression. In frail patients with suitable anatomy, EVAR is then the proposed treatment of choice. The underlying predictive thoughts in this clinical decision are primarily focusing on the impending risk of aneurysm rupture. In a meta-analysis of observational studies, Parkinson et al. found that the annual risk of AAA rupture in frail patients was 3.5% for AAAs of 5.5–6 cm, 4.1% for AAAs of 6.1–7 cm, and 6.3% for AAAs > 7 cm^15^. More recently published data by Lancaster et al.^16^ found annual rupture rates for patients with AAA of only 2.2% for diameters 5.5–6 cm, but 6.0% for diameters 6.1–7 cm, and 18.4% for diameters > 7 cm.

#### Who should be treated and when?

Similar to the VSGNE mortality risk scoring scheme for perioperative risk assessment in patients with asymptomatic AAA^21^, which has found its way into the SVS guidelines^8^, a scoring scheme for life expectancy could help to further improve patient care. A more flexible approach with a tailored decision rather than a fixed threshold diameter is likely to further improve the care of patients with AAA.

The simple and pragmatic risk score shown in Fig. 3 allows identification of a high-risk cohort of patients with asymptomatic AAA who may not benefit from preventive aneurysm repair according to current treatment criteria. In this study, octogenarians with moderately impaired renal function or COPD were "high-risk" patients (score ≥ 19 points) and had an expected 5 years survival of only 40%. It is likely that patients with this risk profile are more likely to benefit from targeted work-up of these comorbidities to avoid associated morbidity than from elective AAA treatment at 5.5 cm diameter. A tailored diameter indication according to the risk profile might reduce the burden on individual patients who might not benefit from aneurysm repair, and conserve health system resources.

On the other hand, patients with a favourable risk profile (score ≤ 8 points) had an excellent 5 years survival rate of 89%. Besides open aneurysm repair, EVAR seems to a valuable treatment option for these low-risk patients and they are likely to benefit from surgical treatment even at the current diameter threshold.

### Limitations

The results of this study seem plausible, as they are consistent with the findings of previous published studies in this area^21,28,30,38,41^. Nevertheless, there are some limitations that need to be considered.

First, the dataset reflects the experience of a single centre. Unmeasured local characteristics of the patient population or patient care could affect the transferability of our results to an external cohort.

Second, it is possible that there are other relevant variables that significantly affect survival after EVAR but were not available for analysis.

Third, the COPD variable was binary. Information on GOLD stage and/or forced expiratory volume could help to further improve this model. In addition, renal function could change over time. No sequential measurements were available that would have allowed adjustments for a time-varying covariate. Further, this model was built and validated on a patient cohort with a median age of 75 (IQR: 70–80) years. Robustness of this model outside this age range is not recommended.

Fourth, women were underrepresented in this cohort, as AAA occurs predominantly in men. Gender was not associated with poorer survival; however, the identified risk factors might have a different impact in women than in men.

Finally, we present a temporal validation on a new cohort of patients treated in the same setting. To minimise the problem of overfitting, bootstrap validation was performed. Nevertheless, a true external validation with an unknown cohort in an unknown setting is the gold standard to test the accuracy of a predictive model.

## Conclusion

Older age, impaired renal function and COPD are independent predictors of poorer survival after EVAR. This predictive model could help support the role of EVAR by distinguish patients with favourable life expectancy who would likely benefit from elective aneurysm repair from patients for whom elective aneurysm repair is not appropriate at the current diameter threshold. However, external validation in a larger cohort is needed before implementation in clinical routine.

## Supplementary Information

Supplementary Information.

## Data Availability

The data that support the findings of this study are available on request from the corresponding author, LM. The data are not publicly available due to their containing information that could compromise the privacy of research participants.
