# Supplementary material for: Prognostic model for survival of patients with abdominal aortic aneurysms treated with endovascular aneurysm repair
Source: Sci Rep. 2022 Nov 15;12:19540. doi: 10.1038/s41598-022-24060-5 (PMC9666454; doi:10.1038/s41598-022-24060-5)
Supplement: Supplementary file 1 — Supplementary Information. [file 41598_2022_24060_MOESM1_ESM.pdf]

Supplementary Table 1: Multivariate cox regression model

**Overall survival**	**2001 - 2012**		**2013 - 2020**	
**Variable**	**n=251**	**HR (95%-CI)**	**n=307**	**HR (95%-CI)**
Age, *years*	74.6 (7.6)	1.09 (1.05-1.14)	75.2 (8.2)	1.11 (1.06-1.16)
Female sex	17(6.8%)	1.09 (0.53-2.23)	32 (10.4%)	0.28 (0.06-1.23)
Arterial hypertension	210 (83.7%)	0.81 (0.44-1.47)	252 (85.1%)	0.68 (0.34-1.39)
Diabetes mellitus	50 (19.9%)	2.94 (1.73-4.99)	59 (19.3%)	2.02 (1.05-3.90)
Dyslipidemia	195 (77.7%)	1.30 (0.78-2.18)	276 (90.2%)	0.45 (0.19-1.05)
BMI, *kg/m²*	27.4 (4.2)	0.95 (0.89-1.01)	27.6 (5.4%)	0.99 (0.92-1.06)
Smoking	191 (76.4%)	1.07 (0.64-1.77)	203 (71.0%)	0.68 (0.36-1.27)
COPD	71 (28.4%)	2.27 (1.41-3.70)	62 (20.3%)	2.13 (1.12-4.04)
eGFR, *ml/min/1.73²*	61.9 (21.4)	0.97 (0.96-0.99)	68.3 (22.1)	0.98 (0.96-1.00)
Creatinine, *µmol/l*	104.7 (61.6)	1.00 (1.00-1.00)	104.2 (65.1)	1.01 (1.00-1.01)
PAD, *none*	178 (70.9%)	ref.	262 (85.6%)	ref.
Fontaine stage 1	28 (11.2%)	1.76 (0.96-3.21)	21 (6.9%)	0.99 (0.29-3.40)
Fontaine stage 2	24 (9.6%)	0.91 (0.43-1.95)	23 (7.5%)	1.83 (0.74-4.56)
Fontaine stage 3	21 (8.4%)	0.86 (0.40-1.84)	0	-
Coronary artery disease	135 (53.8%)	1.40 (0.86-2.30)	175 (57.0%)	1.59 (0.84-3.03)
Myocardial infarction	58 (23.2%)	1.29 (0.85-1.97)	61 (19.9%)	1.72 (0.77-3.85)
Aneurysm diameter, *mm*	57.8 (10.7%)	0.99 (0.97-1.01)	60.2 (11.0%)	1.02 (0.99-1.05)

Footnote:

Completeness of data is reported in Table 1. HR: Hazard ratios derived from multivariate cox regression models. Not having the condition served as the reference group for the factor variables; male sex served as a reference group for the sex variable.

The proportional hazard assumption was tested for both models (scaled Schoenfeld residuals; Global test: *P*=.082 for 2001-2012 model, *P*=.262 for 2013-2020 model). 95%-CI: 95% confidence interval of the HR.

BMI: body mass index; COPD: chronic obstructive pulmonary disease; eGFR: estimated glomerular filtration rate according to the “Modification of Diet in Renal Disease Study” (MDRD) equation presented as mean and standard deviation; PAD = peripheral arterial disease as clinical stage according to the Fontaine classification.

Supplementary Figure 1: Overall Survival by Cohort

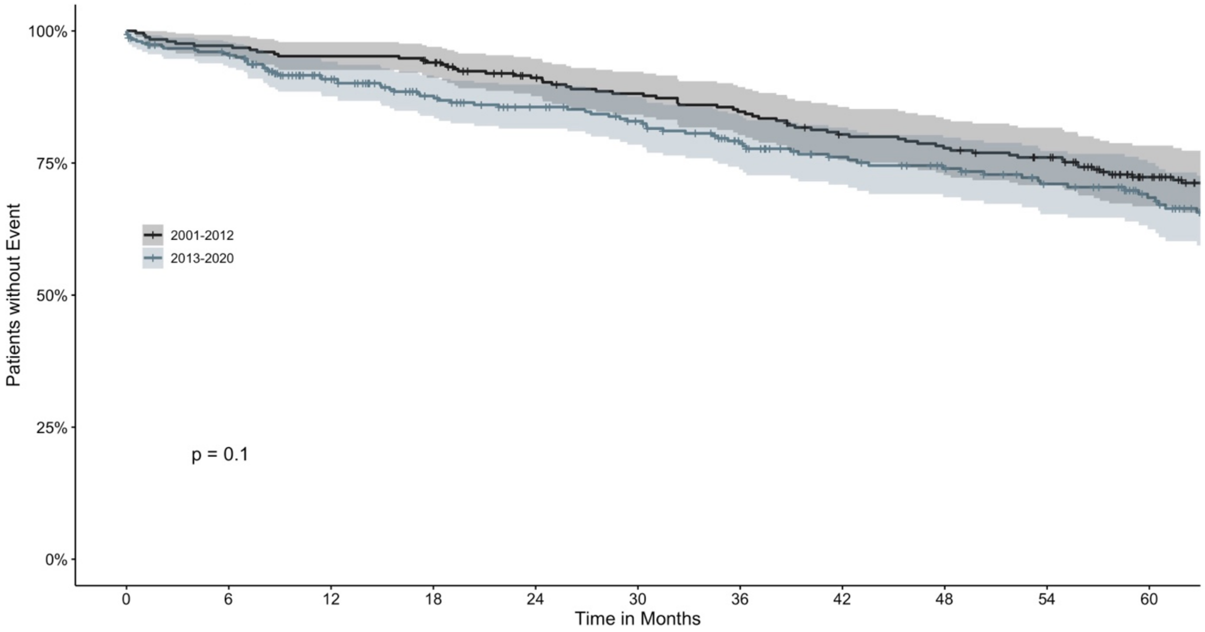

Number at risk: n (%)

	0	6	12	18	24	30	36	42	48	54	60
2001-2012	251 (100)	244 (97)	239 (95)	234 (93)	215 (86)	206 (82)	198 (79)	185 (74)	179 (71)	171 (68)	139 (55)
2013-2020	307 (100)	285 (93)	244 (79)	212 (69)	195 (64)	182 (59)	160 (52)	142 (46)	132 (43)	118 (38)	101 (33)

Time in Months

Cumulative number of events

	0	6	12	18	24	30	36	42	48	54	60
2001-2012	0	7	12	15	22	29	37	47	53	57	65
2013-2020	1	14	27	35	40	46	55	60	64	69	73

Time in Months

<u>Legend:</u>

Kaplan-Meier Estimators with corresponding 95% confidence interval for overall survival stratified by Cohort A and B. Log-rank test was used to compare difference in survival.

Supplementary Figure 2: Overall Survival by Age

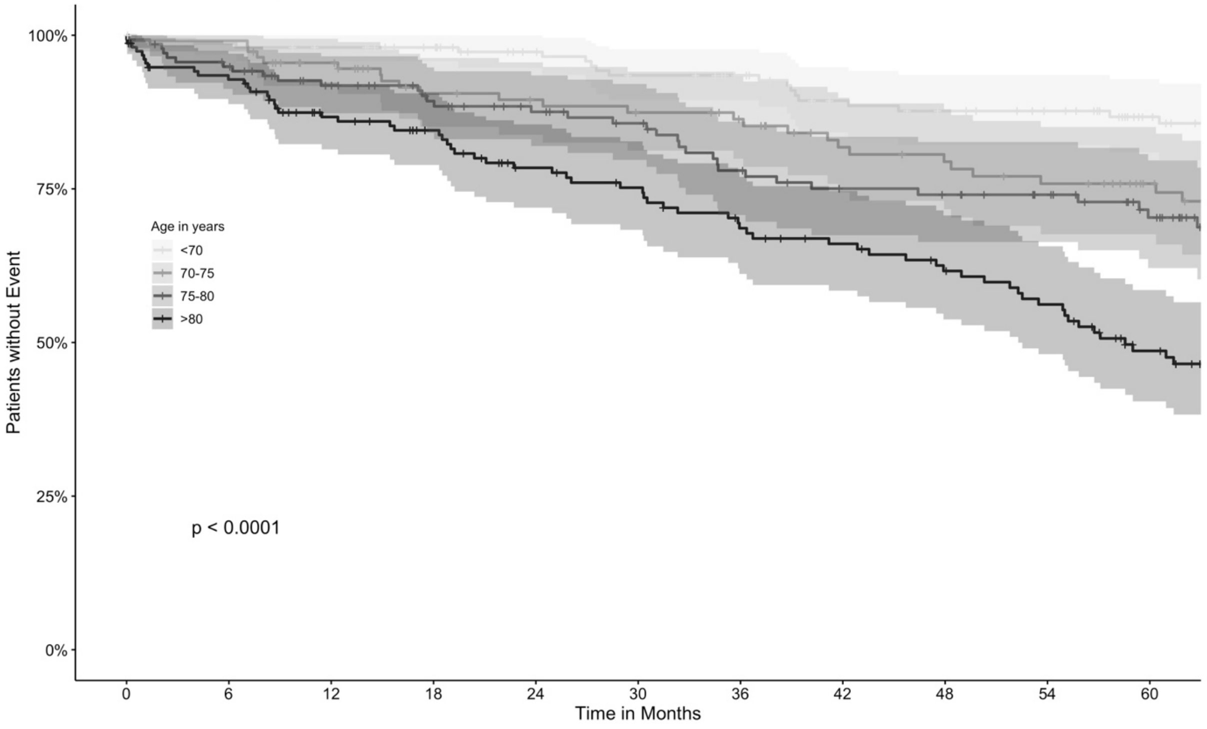

Number at risk: n (%)

Age in years	0	6	12	18	24	30	36	42	48	54	60
<70	153 (100)	149 (97)	146 (95)	140 (92)	129 (84)	122 (80)	116 (76)	106 (69)	102 (67)	97 (63)	84 (55)
70-75	112 (100)	111 (99)	101 (90)	89 (79)	86 (77)	83 (74)	79 (71)	70 (62)	67 (60)	63 (56)	54 (48)
75-80	138 (100)	129 (93)	115 (83)	105 (76)	97 (70)	91 (66)	81 (59)	75 (54)	73 (53)	67 (49)	55 (40)
>80	155 (100)	140 (90)	121 (78)	112 (72)	98 (63)	92 (59)	82 (53)	76 (49)	69 (45)	62 (40)	47 (30)

Time in Months

<u>Legend:</u>

Kaplan-Meier Estimators with corresponding 95% confidence interval for overall survival stratified by age group. Log-rank test was used to compare difference in survival.

Supplementary Figure 3: Overall Survival by COPD

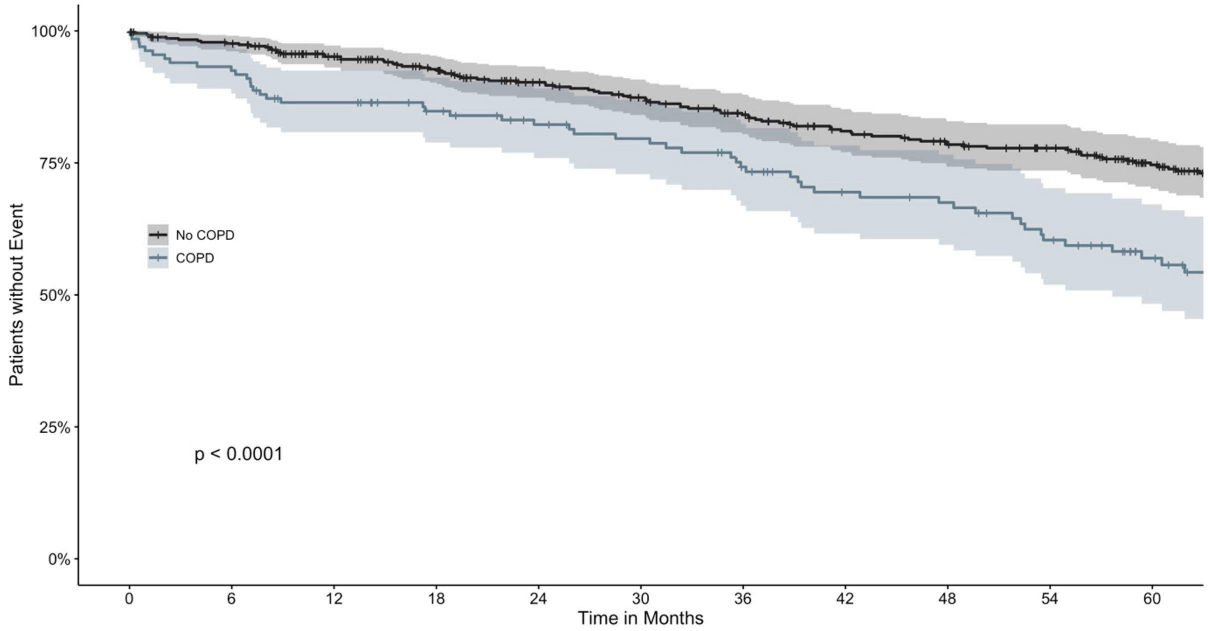

Number at risk: n (%)

Time in Months	0	6	12	18	24	30	36	42	48	54	60
No COPD	423 (100)	405 (96)	370 (87)	343 (81)	315 (74)	298 (70)	277 (65)	256 (61)	243 (57)	230 (54)	195 (46)
COPD	133 (100)	123 (92)	112 (84)	103 (77)	95 (71)	90 (68)	81 (61)	71 (53)	68 (51)	59 (44)	45 (34)

Cumulative number of events

Time in Months	0	6	12	18	24	30	36	42	48	54	60
No COPD	1	10	20	29	38	48	59	69	77	79	88
COPD	0	10	18	20	23	26	32	37	39	46	49

<u>Legend:</u>

Kaplan-Meier Estimators with corresponding 95% confidence interval for overall survival stratified by COPD (chronic obstructive pulmonary disease). Log-rank test was used to compare difference in survival.

Supplementary Figure 4: Overall Survival by Kidney Function (eGFR)

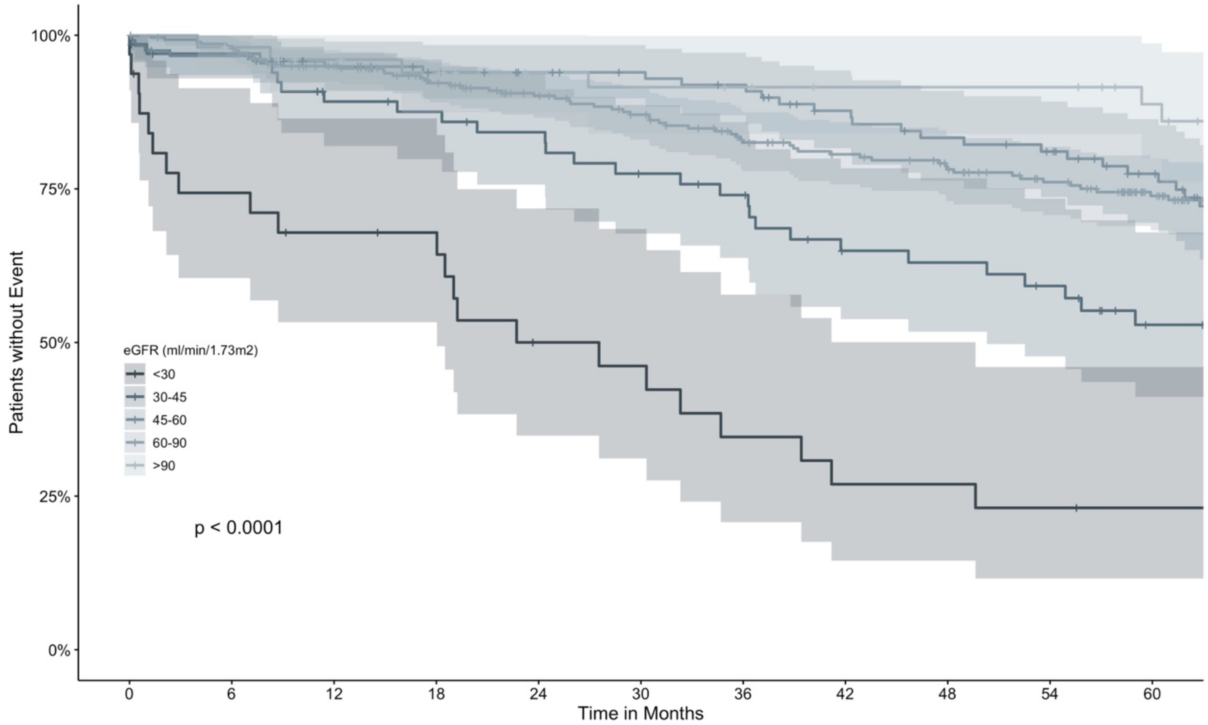

Number at risk: n (%)

eGFR (ml/min/1.73m2)	0	6	12	18	24	30	36	42	48	54	60
<30	32 (100)	23 (72)	20 (62)	19 (59)	13 (41)	12 (38)	9 (28)	7 (22)	7 (22)	6 (19)	5 (16)
30-45	67 (100)	63 (94)	55 (82)	53 (79)	50 (75)	45 (67)	42 (63)	34 (51)	33 (49)	30 (45)	22 (33)
45-60	120 (100)	115 (96)	105 (88)	99 (82)	95 (79)	92 (77)	89 (74)	80 (67)	75 (62)	71 (59)	61 (51)
60-90	284 (100)	275 (97)	253 (89)	228 (80)	209 (74)	197 (69)	177 (62)	167 (59)	157 (55)	143 (50)	118 (42)
>90	51 (100)	49 (96)	46 (90)	44 (86)	40 (78)	39 (76)	38 (75)	36 (71)	36 (71)	36 (71)	32 (63)

Time in Months

<u>Legend:</u>

Kaplan-Meier Estimators with corresponding 95% confidence interval for overall survival stratified by kidney function (eGFR = estimated glomerular filtration rate). Log-rank test was used to compare difference in survival.
